# Patterns of equipment use for autistic children in multi-sensory environments: Time spent with sensory equipment varies by sensory profile and intellectual ability

**DOI:** 10.1177/13623613231180266

**Published:** 2023-07-07

**Authors:** Katy L Unwin, Georgina Powell, Alice Price, Catherine RG Jones

**Affiliations:** 1 Cardiff University, UK; 2 La Trobe University, Australia

**Keywords:** autism, motivation, multi-sensory environment, preference, sensory profile and sensory behaviours, sensory room

## Abstract

Multi-sensory environments are widely used with autistic children. However, there are no data on how autistic children choose to spend time in the room and how this relates to their sensory, behavioural and intellectual profiles. We observed the frequency and duration of visits to multi-sensory environment equipment of 41 autistic children during 5 min of free play. At a group level, the bubble tube and touch, sound and light board were both highly popular, with the fibre optics and tactile board receiving less attention. Sensory seeking behaviours were more commonly observed in the multi-sensory environment than sensory-defensive behaviours. We found that observed sensory seeking behaviours, along with parent-reported sensory behaviours and non-verbal intelligence quotient, were associated with specific patterns of multi-sensory environment equipment use, but broader autistic behaviours were not. These data suggest that the multi-sensory environment equipment preferences of autistic children are related to individual differences in sensory behaviour and non-verbal intelligence quotient. This information has the potential to inform the development of best-practice multi-sensory environment guidelines that focus on individual needs.

**Lay abstract:**

Multi-sensory environments, often called sensory rooms, are widely used with autistic children. However, we know very little about how autistic children choose to spend their time in multi-sensory environments. We also do not know how their equipment preferences relate to their individual characteristics such as their sensory differences, level of ability or general autistic behaviours. We measured the frequency and duration of visits to multi-sensory environment equipment of 41 autistic children during 5 min of free play. The bubble tube and touch, sound and light board were both highly popular, with the fibre optics and tactile board receiving less attention. The children displayed significantly more sensory seeking behaviours in the multi-sensory environment than sensory-defensive behaviours. These sensory seeking behaviours, as well as the sensory behaviours that their parents reported they showed in daily life, were associated with specific patterns of multi-sensory environment equipment use. Non-verbal ability was also associated with multi-sensory environment equipment use, but broader autistic behaviours were not. Our findings show that the multi-sensory environment equipment preferences of autistic children are related to individual differences in sensory behaviours and non-verbal ability. This information could be useful for teachers and other practitioners who want to know how best to use multi-sensory environments with autistic children.

## Introduction

Multi-sensory environments (MSEs, also called sensory or Snoezelen^®^ rooms) are specialised spaces containing sensory equipment that provide a variety of sensory input across modalities. They are widely used in special schools for autistic children and recommended as part of best practice within education (Altenmüller-Lewis, 2017; Department for Education, 2015). Despite their widespread use, there are no established and empirically driven guidelines to support practitioners using MSEs with pupils, and very little empirical research that has specifically considered the experiences of autistic children (Kim & Park, 2022; Mey et al., 2015; Unwin et al., 2021b).

MSEs vary in their contents, with a large variety of equipment available that differ in the modalities of stimulation, as well as the level of engagement necessary to produce stimulation. For example, a bubble tube is a cylindrical tube containing bubbling water that is illuminated by colour-changing lights. It requires no active engagement for sensory stimulation and is highly dynamic, producing visual (i.e. bubbles and colour changes) and auditory (i.e. buzzing of the motor) stimulation. The tube also provides tactile (i.e. vibrations from the motor) stimulation when touched. By contrast, a tactile board, which is typically wall-mounted and contains a variety of textured surfaces, requires the user to actively engage to receive tactile stimulation. Although tactile boards are typically colourful and engagement can produce sounds (e.g. knocking the board), they are not electronic, thus provide more muted stimulation. Faced with such contrasting equipment, it can be difficult for practitioners to make informed decisions about what equipment to purchase (Carter Stephenson, 2012) and to decide how to use it with pupils (Unwin et al., 2021a).

Practitioners agree that more guidance and training are needed to support working with autistic children in MSEs (Unwin et al., 2021a). Where school policies and guidance exist, they tend to focus on how to use the equipment rather than pedagogical considerations (Carter & Stephenson, 2012). Alongside the need for better guidance, practitioners have also stressed the importance of centring the MSE use on the child’s needs (Unwin et al., 2021a). Therefore, one way of supporting practitioners is to better understand the patterns of engagement that autistic children show towards different types of MSE equipment. Practitioners have described how children with special educational needs, including autism, will sometimes actively seek out specific sensory equipment within the MSE (Stephenson & Carter, 2011b). However, most investigations of MSE use, including those with autistic participants (Kim & Park, 2022; Mey et al., 2015; Novakovic et al., 2019; Unwin et al., 2021b), have focused on behavioural outcomes and do not explore patterns of use or preference.

Currently, there are no data informing practitioners about what equipment within MSEs are used most frequently by autistic children, and how these perceived preferences might relate to individual differences in sensory, cognitive and behavioural profiles. There is wide heterogeneity within the autistic population, and autistic children with different sensory profiles and needs (DeBoth et al., 2023; Lane et al., 2014) may seek out different equipment within the MSE. It is also unclear whether other individual characteristics such as intellectual ability, age or broader autistic behaviours would affect engagement. The current study used naturalistic observation to investigate the pattern of equipment use among 41 autistic children using an MSE. During a 5-min 1:1 free play session with an experimenter, where the child had full autonomy over how and where they spent their time, we recorded the first piece of equipment that the child used as well as the frequency of visits and the time spent at each piece of equipment. To investigate individual differences in patterns of use, we also explored how sensory profiles, measured both through parent-report (Sensory Profile [SP]; Dunn, 1999) and observed sensory behaviours within the room, associated with the time spent at the different pieces of equipment. We additionally explored the extent to which time spent at equipment correlated with age, broader autistic behaviours (Social Communication Questionnaire (SCQ); Berument et al., 1999) and non-verbal intelligence quotient (NVIQ).

## Methods

### Participants

As part of a larger MSE study, 41 autistic children (8 female) aged 4–12 years (*M* = 8 years, standard deviation (*SD*) = 2.05 years) participated. All had a clinical autism diagnosis, with confirmatory Autism Diagnostic Observation Schedule, Second Edition (ADOS-2; Lord et al., 2012) assessments obtained for 40 participants (for further details of inclusion criteria and recruitment see Unwin et al., 2021b). Their mean score on the SCQ (Berument et al., 1999) was *M* = 24.8 (*SD* = 6.48). Nineteen (46%) had fluent speech, the remaining 54% were either pre-verbal or only spoke only single words (*n* = 19) or had phrase speech (*n* = 3).

Ability was measured using the Wechsler Abbreviated Scale of Intelligence, Second Edition (WASI-II; Wechsler, 2011) and the Wechsler Preschool and Primary Scale of Intelligence, Fourth Edition (WPPSI-IV; Wechsler, 2012), depending on the participants’ age (i.e. >6 years = WASI, *n* = 27; <6 years = WPPSI, *n* = 8). The amount of data collected was affected by the verbal abilities of the participants as well as ability to engage with the assessments. In total, cognitive ability data were collected for 35 participants but with fewer participants accessing the verbal intelligence quotient (IQ) measure (Table 1). As such, NVIQ was used for analysis.

**Table 1.** Cognitive ability data (*n* = 35) from the Wechsler Abbreviated Scale of Intelligence and Wechsler Preschool and Primary Scale of Intelligence assessments.

	*n*	Minimum–maximum	*M*	*SD*
Verbal intelligence quotient (VIQ)	19	59–126	94.32	19.74
Non-verbal intelligence quotient (NVIQ)	35	46–142	90.43	24.23
Full-scale intelligence quotient (FSIQ)	19	69–128	96.95	15.75

*SD*: standard deviation.

Data were not collected on participant race/ethnicity or socioeconomic status. Ethical approval for this study was granted by the School of Psychology Ethics Committee, Cardiff University, with parents providing written informed consent for their child’s participation.

### Materials and procedure

**Figure 1.**
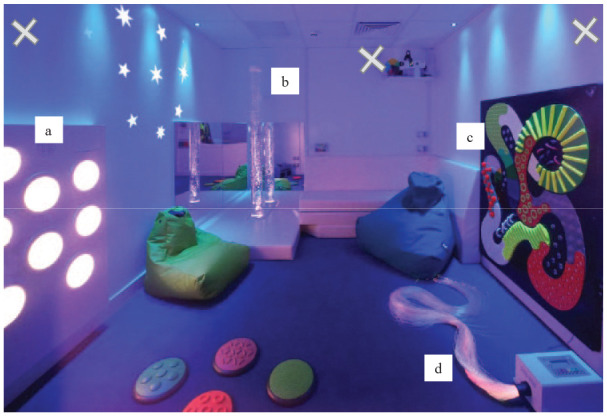
Image of the multi-sensory environment used in this study containing, (a) touch, sound and light board, (b) bubble tube, (c) Tactile board and (d) fibre optics. ‘X’ marks camera positioning, with all cameras pointing out and down. NB. The projected wall stars and textured floor dots were not included for the MSE sessions in this study. Source: Photo courtesy of Mike Ayres.

The study took place in a purpose-built MSE (Figure 1) where the participants could freely engage with equipment for 5 min. The equipment included in the MSE was informed by equipment commonly used in MSEs in UK special schools (Unwin et al., 2021a), as well as a survey of relevant literature. We focussed on the four primary pieces of equipment in the room: touch, sound and light board (TSLB), bubble tube, tactile board and fibre optics (Table 2).

**Table 2.** Description of multi-sensory environment equipment used in the study.

Item & description of primary sensory features	Modalities of stimulation			Description of primary sensory stimulation	Engagement required for stimulation		
	Auditory	Visual	Tactile		Auditory	Visual	Tactile
a) Touch, sound and light board: Board with eight buttons; when touched they produce a coloured light and a sound.	✓E	✓E	✓	Eight buttons can be pressed on the equipment lighting up one of eight colours and playing a piano note. Buttons can be pressed concurrently.	✓	✓	✓
b) Bubble tube: Colour changing water-filled tube with bubbles that travel upwards continuously.	✓E	✓E	✓	One of eight alternating colours are presented every 3 s.	✗	✗	✓
c) Tactile board: Colourful wall-mounted board containing a variety of textures.	✓	✓	✓	Variety of textures can be touched using body parts.	✓	✗	✓
d) Fibre optics: Fibre optic cabling that lights up.	✓	✓E	✓	One of eight alternating colours are presented every 3 s.	✓	✗	✓

E: electronic.

(1) For ‘Modalities of stimulation’, the perceived primary modality of stimulation has been shaded in grey. If the stimulation of the auditory or visual modalities were primarily electrical (lights or sounds) then an ‘E’ is included. (2) For ‘Engagement required for stimulation’, we refer to whether the equipment needed to be touched for the specified modality to be stimulated. A ✓indicates that touched was required; ✗indicates that the modality was passively stimulated (e.g. the humming of the bubble tube was audible whether or not it was touched).

The free play session took place after the participant had spent two blocks of 15 min in the MSE (see Unwin et al., 2021b for details of this separate study), with a break being provided if required. For context, in the previous two blocks of 15 min, the participant used the MSE with the experimenter, in one condition they had control over the sensory changes in the room and in the other condition the sensory changes were automatic. In these previous blocks, the participant was specifically directed to one piece of sensory equipment at a time, with only the TSLB, bubble tube and fibre optics from the current study included. For the free play session used in the current study, the participant was given the instruction, ‘You can play’. If the participant did not independently engage with any equipment, a piece of equipment was demonstrated for them, along with verbal encouragement. If necessary, this process was completed twice. All participants engaged with at least one piece of equipment. As the session was designed to mimic a free play session, experimenter responses were natural and not limited. The session was video recorded using three cameras covering all areas of the room (see Figure 1). Following the session, videos were exported, synced and loaded into ELAN (2018) for coding of sensory behaviours.

A sensory observation coding scheme (see Unwin et al., 2021b) captured the frequency and duration (s) of sensory seeking and sensory-defensive behaviours produced in the auditory, visual and tactile modalities. The coding scheme followed best practice guidelines for item development (Boateng et al., 2018), including identification of domains and item generation through deductive methods. Relevant items and corresponding definitions were extracted from a range of sources, including validated sensory and autism measurement tools. These included observation (Sensory Processing Assessment, Baranek, 1999b; ADOS-2, Lord et al., 2012; Sensory Assessment for Neurodevelopmental Disorders (SAND), Siper et al., 2017) and questionnaire (Sensory Experiences Questionnaire, Baranek, 1999a; SP, Dunn, 1999; Glasgow Sensory Questionnaire, Robertson & Simmons, 2013) measures. Sensory seeking behaviours were coded when the participant sought to reinforce or intensify their sensory experience (e.g. Baranek et al., 2018). For example, the seeking visual code, ‘Unusual visual inspection’ included looking/peering at an object for a prolonged period or at an unusual angle or proximity (adapted from Baranek et al., 2018; SAND). Conversely, defensive behaviours (akin to hyper-responsivity) were coded when the participant actively tried to reduce stimuli, defending themselves by retracting or pulling away from stimulation. For example, the defensive auditory code, ‘Putting hands (or hand) over, or finger/s in, ears’ (Lane et al., 2010; ADOS-2; SAND). The coding scheme is available upon request.

The frequency and duration (s) of visits to the four pieces of equipment, along with the equipment a participant chose to visit first was additionally coded. Where the child was given a prompt towards their first piece of equipment, their data were not included in first visit analysis. A visit to a piece of equipment was coded when the child began engaging with the equipment (e.g. touched a button on the TSLB; fixated their gaze on the bubble tube), and these visits to each piece of equipment were then tallied to provide the frequency of visits. The duration of the visit was timed from the moment that the child engaged with the equipment, until they stopped engaging with it.

### Parent-report measures

The SCQ (Berument et al., 1999) is based on the Autism Diagnostic Interview-Revised (Lord et al., 1994) and was included as a general measure of autistic behaviours. It included 40 items in which a parent responds (yes/no) whether the behaviour is present for their child. Scores range from 0 to 39, with more autistic behaviours being represented by a higher score. The SCQ has excellent reliability and good validity (Berument et al., 1999).

The SP (Dunn, 1999) is a 125-item questionnaire that captured the frequency of sensory behaviours a child performs in daily life in response to sensory input from ‘Never’ (5 points) to ‘Always’ (1 point). A quadrant of scores are produced representing high or low neurological thresholds for sensory stimuli and passive or active self-regulation strategies. A higher score indicated less endorsement, thus a low score is synonymous with unusual sensory patterns of responding. A low ‘Registration’ score reflected a high neurological threshold (i.e. more stimulation required to register the input) and a passive self-regulation strategy (i.e. missing sensory input). A low ‘Seeking’ score reflected a high neurological threshold and an active self-regulation strategy (i.e. seeking out sensory input). A low ‘Sensitivity’ score reflected a low neurological threshold (i.e. less stimulation required to register the input) and passive self-regulation strategy, while an ‘Avoiding’ score reflected a low neurological threshold, but an active self-regulation strategy such as withdrawal. These quadrants were composed of a different number of items: registration (score range = 15–75), seeking (26–130), sensitivity (20–100) and avoiding (29–145). Reliability for the different quadrants ranged from acceptable to excellent, with generally good validity (Dunn, 1999; Ohl et al., 2012).

### Analyses

Twenty-five percent of the free play videos were double coded to calculate inter-rater reliability, with good reliability for frequency (intraclass correlation (ICC) = 0.86, *p* < 0.01), and excellent reliability for the duration of sensory seeking behaviours (ICC = 0.98, *p* < 0.001).

Missing data were identified for the SP and ranged from 0% to 17% across quadrant score items. The number of participants with incomplete data on each quadrant was between three (Registration) and 11 (Avoiding). Little’s Missing Completely at Random test (Little, 1988) was non-significant, and variables such as age, gender and NVIQ did not predict missingness (*p* > 0.05), suggesting participants with missing data were not dissimilar to those without. We therefore applied multiple imputation using predictive mean matching, where k = 3 and number of imputations = 40. Quadrant totals, and their associated modality subscales, were subsequently calculated using imputed values (Enders, 2013; Heymans & Eekhout, 2019; van Buuren, 2012). This approach is suitable for non-normal data and small sample sizes (Kleinke, 2018; van Ginkel et al., 2020) and has additional benefit over traditional approaches (e.g. listwise deletion) as it prevents loss of information (Heymans & Eekhout, 2019).

Variables were assessed for normality of distribution through visual inspection, the Shapiro–Wilk test, and skewness and kurtosis, with non-parametric tests where appropriate. Repeated measures analyses of variance (ANOVAs) and paired sample t-tests explored the pattern of the duration and frequency of visits to the MSE equipment and the observed seeking and defensive-sensory behaviours within the MSE. Correlations explored the association between the SP and observed sensory behaviours on time spent at the equipment. Additional correlations explored other individual differences (age, IQ and broader autistic behaviours). Partial correlations were conducted where relevant to explore possible shared variance.

### Community involvement statement

The study was informed by interviews with educational practitioners working with autistic children (Unwin et al., 2021a). However, autistic people and their families were not involved in the development of the study.

## Results

### Patterns of equipment engagement

**Figure 2.**
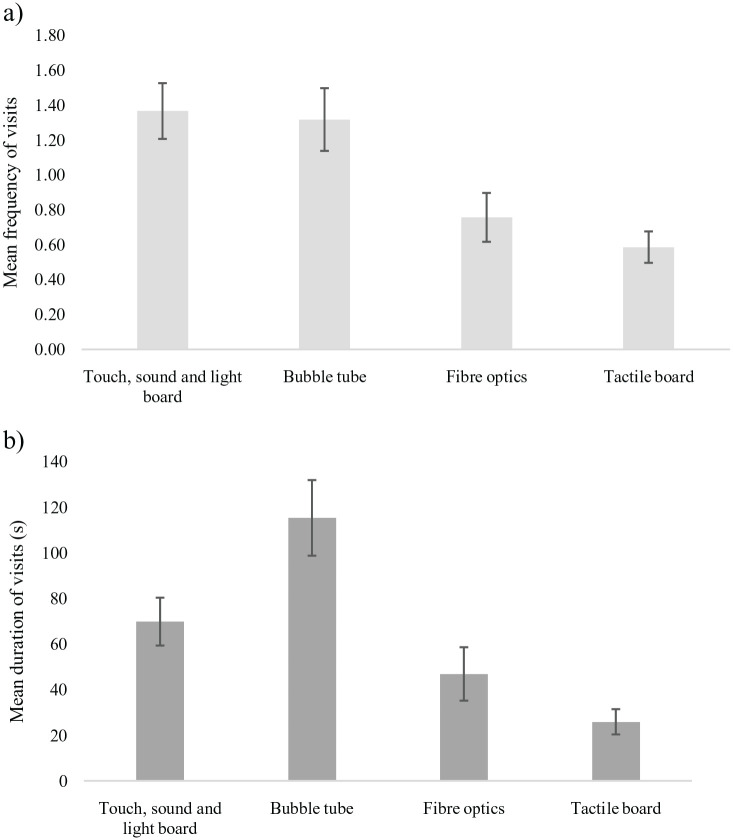
(a) Mean frequency and (b) mean overall duration of visits to each piece of equipment in the MSE during the five-minute free play session. Error bars represent standard errors.

Across the five-minute free play session, participants engaged with the four pieces of equipment for an average of 4.3 min (*SD* = 58 s), reflecting 86% of the session time. On average they made 4.02 (*SD* = 2.50) visits to the equipment, including revisits (Figure 2). Twenty-one participants (51%) did not require prompts to first engage with the equipment. Of these 21 participants, 13 (48%) visited the bubble tube first, 10 (37%) visited the TSLB first, 3 (11%) visited the fibre optics first, while only 1 (4%) chose the tactile board first.

Both the frequency (*F*(3, 120) = 10.09, *p* < 0.001, η_p_^2^ = 0.20) and duration (*F*(1.91, 76.45) = 8.29, *p* < 0.001, η_p_^2^ = 0.17) of visits to the four pieces of equipment differed significantly, with post hoc comparisons included in Table 3. The bubble tube and TSLB were visited, on average, more than once each. Participants spent more time at the bubble tube, but this difference was not significant. The overall pattern of post hoc comparisons indicated that the bubble tube was marginally more popular than the TSLB. Correlations between the two indices of engagement showed that frequency and duration were highly correlated at each piece of equipment (*r_s_* = 0.61–0.90, *p* < 0.01).

**Table 3.** Post hoc comparisons of the frequency and duration of visits to the four pieces of equipment.

Equipment comparisons	Frequency of visit (mean difference)	Duration of visit (mean difference)
Touch, sound and light board		
Versus bubble tube	.05	–45.44
Versus fibre optics	.61**	22.95
Versus tactile board	.78***	43.93***
Bubble tube		
Versus fibre optics	.56*	68.39*
Versus tactile board	.73*	89.37***
Fibre optics		
Versus tactile board	.17	20.98

* *p* < 0.05; **p < 0.01; ***p < 0.001.

### Patterns of sensory behaviours: Parent self-report and observed

Descriptives were examined for the SP quadrant scores and the observed sensory behaviours (Table 4).

**Table 4.** Descriptives for the quadrant scores of the Sensory Profile (SP; Dunn, 1999) and the frequency and duration (s) of observed seeking and defensive-sensory behaviours.

	Mean (*SD*)	Range (Minimum–maximum)
SP quadrant scores		
Registration	49.66 (13.16)	15–69
Avoiding	89.17 (19.07)	40–126
Seeking	80.21 (16.98)	41–115
Sensitivity	62.95 (13.72)	34–87
Observed-sensory behaviours: Frequency		
Seeking	12.68 (9.22)	0–40
Defensive	1.39 (2.33)	0–9
Observed-sensory behaviours: Duration (s)		
Seeking	89.80 (91.07)	0–373.73
Defensive	4.08 (9.34)	0–38.33

*SD*: standard deviation.

For the SP Registration, Avoiding and Sensitivity quadrants, the mean participants’ score was lower than 2 *SD* below the mean of a normative sample of 3- to 10-year olds (Dunn, 1999). For Seeking, the mean participants’ score was lower than 1 *SD* below the normative mean, but not more than 2 *SD* from the mean.

For the observed behaviours, sensory-defensive behaviours were less frequent (*t*(40) = 7.52, *p* < 0.001, *d* = 1.18) and shorter in duration (*t*(40) = 6.05, *p* < 0.001, *d* = 0.95) than sensory seeking behaviours. Twenty-two (54%) of the sample produced no defensive behaviours, compared to only one child (2%) who produced no sensory seeking behaviours. The correlation between the frequency and duration of the observed sensory seeking behaviours indicated a large effect (*r_s_* = 0.79, *p* < 0.001). This strong correlation, along with the strong correlations between the duration and frequency of visits to the different MSE equipment, meant that our additional analyses focussed on duration of behaviours and visits only.

### Correlations between time spent at equipment and parent-reported and observed sensory behaviours

The SP quadrant scores and duration of sensory seeking observed behaviours were correlated with duration spent at each piece of equipment (Table 5).

**Table 5.** Spearman’s Rho correlations between the duration (s) of time spent at the equipment and (1) Sensory Profile (SP; Dunn, 1999) quadrant scores and (2) the duration of observed sensory seeking behaviours in the session. NB. Lower SP scores indicate more sensory behaviours.

	df	Touch, sound and light board	Bubble tube	Fibre optics	Tactile board
SP quadrant scores					
Registration	39	−.23	.15	−.02	−.33*
Avoiding	39	−.31*	.25	.003	−.40**
Seeking	39	.07	.08	−.01	.02
Sensitivity	39	−.30	.31*	−.02	−.48**
Observed-sensory behaviours: Duration (s)					
Seeking	39	−.60***	.76***	−.26	−.45**

* *p* < 0.05; **p < 0.01; ***p < 0.001.

Focussing first on the SP, caution is required when interpreting multiple correlations, particularly as effects were often small and only a minority of correlations reached significance. However, a distinct pattern was clear across the four pieces of equipment. More time spent at the TSLB and the tactile board generally associated with more parent-reported sensory behaviours, while more time spent at the bubble tube generally associated with fewer parent-reported sensory behaviours. In contrast, there was a negligible association between time spent at the fibre optics and parent-reported sensory behaviours. It is also notable that the pattern of effects were found across Registration, Avoiding and Sensitivity, with no meaningful associations with Seeking. Post hoc analyses explored the significant correlations for Registration, Avoiding and Sensitivity to see if these were driven by particular modalities (see Supplementary Materials 1).

For the observed sensory seeking behaviours (Table 5), more time spent with the TSLB and the tactile board associated with significantly less time engaging in sensory seeking behaviours. In contrast, more time at the bubble tube was associated with significantly more sensory seeking behaviours.

### Correlations between time spent at equipment and NVIQ, age and autistic behaviours

The duration spent at each piece of equipment was correlated with NVIQ, age and parent-reported autistic behaviours (Table 6).

**Table 6.** Spearman’s Rho correlations between the duration (s) spent at each piece of equipment and NVIQ, age and autistic behaviours (SCQ; Berument et al., 1999).

	Touch, sound and light board	Bubble tube	Fibre optics	Tactile board
NVIQ	.37*	−.50**	.06	.45**
Age (years)	.12	−.20	.08	.42**
Autistic behaviours (SCQ)	−.29	.17	−.02	−.11

NVIQ: non-verbal intelligence quotient; SCQ: Social Communication Questionnaire.

* *p* < 0.05; **p < 0.01.

For NVIQ, more time spent with the TSLB and the tactile board was significantly associated with a higher NVIQ, while more time spent with the bubble tube was significantly associated with a lower NVIQ. There was more limited evidence of the influence of age on time spend at equipment and no clear evidence of an association with broader autistic behaviours.

Finally, we explored whether the significant associations between time spent at equipment and NVIQ and age partially explained the significant effects found between sensory behaviours and time spent at the sensory equipment (Table 7).

**Table 7.** Partialling out (Spearman’s Rho partial correlations) NVIQ and age from the significant correlations between the duration (s) spent at each piece of equipment and sensory behaviours.

Partialled variable	Touch, sound and light board	Bubble tube	Tactile board
	NVIQ	NVIQ	NVIQ + Age
SP Registration	—	—	−.24
SP Avoiding	−.09	—	−.22
SP Sensitivity	—	.18	−.36*
Observed sensory seeking behaviours (duration (s))	−.52**	.62***	−.24

NVIQ: non-verbal intelligence quotient; SP: Sensory Profile (Dunn, 1997).

* *p* < 0.05; **p < 0.01; ***p < 0.001.

For the parent-reported sensory behaviours, only the association between time spent at the tactile board and sensory sensitivity remained significant. For the observed sensory seeking behaviours, the correlations with time spent at the TSLB and the bubble tube remained significant. To further aid interpretation, SP scores and observed sensory behaviours were correlated with NVIQ. Only observed sensory seeking behaviours correlated with NVIQ (see Supplementary Materials 2).

## Discussion

We explored how 41 autistic children with a range of intellectual ability engaged with an MSE during a five-minute free play session. Differentiation was found in the choices that children made about how to spend their time. The bubble tube and TLSB were both highly popular, with the fibre optics and tactile board being engaged with less often. We also found that parent-reported sensory behaviours and observed sensory seeking behaviours, along with NVIQ, were associated with the way autistic children engaged with MSE equipment. This is the first study of its kind to investigate how autistic children choose to use MSEs and how patterns of use associate with their sensory and intellectual profiles.

A notable feature of our findings was that the observed sensory-defensive behaviours performed within the free play session were seen significantly less frequently and for a shorter overall duration than the sensory seeking behaviours. In particular, over half the participants performed no defensive behaviours at all, compared to just 2% showing no seeking behaviours. Defensive behaviours reflect an aversive reaction to sensory stimulation. In contrast, seeking behaviours reflect an excessive interest in a stimulus (American Psychiatric Association, 2013), with sensory fascination described in positive terms by autistic people (Sibeoni et al., 2022). Although the specific characteristics of our MSE limit generalisability, the findings reflect educators’ descriptions of MSEs as a primarily positive sensory space for autistic children (Carter & Stephenson, 2012; Stephenson & Carter, 2011b; Unwin et al., 2021a). A caveat is that autistic adults have also described sensory seeking as a way of avoiding aversive sensory input, or self-soothing and inducing calm when distressed (MacLennan et al., 2022). Thus, it cannot be discounted that, at least for some children, sensory seeking behaviours in the MSE reflect a coping strategy rather than a more straightforward pursuit of sensory pleasure.

We found a hierarchy of equipment preference among our participants. The bubble tube and TSLB were the most popular items, with the bubble tube being marginally more preferred. This overall preference for very stimulating equipment aligns with evidence from Sautter et al. (2008), who found that a group of autistic children preferred to spend time with highly sensory stimulating toys over traditional toys that are typically found in classrooms, such as a bat and ball and dinosaurs. In our study, the two most popular items had clear electronic or mechanical lights and sounds, providing strong auditory and visual sensory saliency. The least popular items, the fibre optics and tactile board, were less stimulating at face value, with neither making an electronic or mechanical sound, and the tactile board not including any electronic component.

### Parent-reported sensory sensitivity and MSE equipment use

Higher parent-reported sensory avoidance was associated with significantly more time spent at both the tactile board and the TSLB. In contrast, higher levels of sensory avoidance were associated with less time at the bubble tube, albeit with a small to medium effect (*r* = 0.25) that was not significant. Similarly, higher parent-reported sensory sensitivity was associated with significantly more time spent at the tactile board and with significantly less time at the bubble tube. The association between higher sensory sensitivity and significantly more time spent at the TSLB was medium sized (*r* = −0.30) but not significant. Both avoiding and sensitivity behaviours reflect a low threshold for detecting sensory changes in the environment. However, whereas avoiding behaviours are defined by an active self-regulatory response, such as withdrawal and avoidance, sensitivity behaviours involve a passive self-regulation strategy (Dunn, 1999).

Although there should be caution regarding the two correlations that were non-significant, the pattern of findings suggest a dissociation where the tactile board and TSLB were visited for longer by children with a low threshold for detecting sensory changes, while the bubble tube was visited less. Furthermore, this was true regardless of whether the child was passive or active in their sensory self-regulation. The tactile board and TSLB only produced their intended sensory effects when touched, unlike the bubble tube where the continual visual and auditory stimulation did not require active engagement. One interpretation is that the tactile board and TSLB are attractive to hypersensitive children because they have greater control over their sensory input, which may in turn make sensory experiences more pleasurable. Indeed, hypersensitivity has been associated with fascinating and pleasurable experiences by autistic adults (Smith & Sharp, 2013) and having control over stimulation has been described by autistic adults as the mediator between sensory comfort and discomfort (e.g. Robertson & Simmons, 2015). The tactile board was non-electronic and therefore perhaps particularly appealing for ‘Sensitive’ children who have difficultly actively regulating sensory input. An additional consideration is that although the association between sensitivity and time spent at the tactile board remained significant, the other correlations reduced to non-significant when NVIQ was controlled. Although NVIQ did not directly correlate with parent-reported sensory behaviours, the pattern of findings should also be considered in the context of non-verbal ability. Particularly, non-verbal ability may mediate the association between sensory profile and equipment choice.

Higher parent-reported sensory registration was also associated with significantly more time spent at the tactile board, with the pattern of associations for the TSLB and bubble tube echoing that found for avoidance and sensitivity, albeit not at a significant level. Registration behaviours reflect a high threshold for detecting sensory input and a passive self-regulation strategy, meaning sensory input can be missed. Registration was the quadrant with the fewest items and only contained two items that directly mapped onto the auditory, visual and tactile modalities. Just over half the items captured the modulation of sensory input that related to the ability to sustain performance, including endurance (e.g. ‘Tires easily) and tone (e.g. ‘Weak muscles’) (Dunn, 1999). It is possible that children who were less sensitive to sensory input and were not driven or able to self-regulate were most comfortable with the tactile board, which is the most sensorily undemanding piece of equipment.

### Observed sensory seeking behaviours and MSE equipment use

A similar dissociation between the time spent at the TSLB and tactile board compared to the bubble tube was found when correlating with the sensory seeking behaviours that the children produced in the room. More time spent at the TSLB and tactile board was associated with less time engaging in sensory seeking behaviours, while more time spent at the bubble tube was associated with more time engaging in these behaviours. One interpretation is that the affordances of the equipment elicited the sensory seeking behaviours. The auditory and visual stimulation provided by the bubble tube was continual. In contrast, the TSLB and the tactile board were inactive, beyond the colourful display of the tactile board, unless being touched. Thus, they might not trigger sensory seeking behaviours to the same degree. Alternatively, the association could be child driven, with children who were naturally sensory seeking being drawn to the bubble tube with its array of overt possibilities for stimulation. The TSLB and tactile board are also strongly under the child’s control, compared to the passive stimulation of the bubble tube, which aligns with our previous research finding that sensory seeking behaviours are reduced when an autistic child has control of the sensory equipment (Unwin et al., 2021b).

The associations between time spent at the MSE equipment and the observed sensory seeking behaviours were not replicated with the SP sensory seeking quadrant. However, the observation measure recorded behaviours across auditory, visual and tactile modalities, whereas the SP quadrant was much broader and included vestibular, multisensory and oral sensory modalities, as well as items relating to sensory modulation. Furthermore, parent report and observations are often poorly correlated (e.g. Lemler, 2012), which in the present study could relate to the timeframe in which information was gathered (i.e. lifetime vs 5 min), or difference in settings (i.e. daily life across multiple settings vs experimental context in a single setting).

### Other individual differences and MSE equipment use

The TSLB and tactile board were engaged with for significantly longer by participants with higher NVIQ, who also spent significantly less time with the bubble tube. Individuals with higher visuo-spatial and object manipulation skills may prefer the intellectual stimulation provided by equipment that requires active manipulation. In contrast, the bubble tube places little demand on the user for its effects to be experienced. It is also the most visually stimulating of the equipment, which is similar to the preference for visual MSE equipment among adults with intellectual disability (Matson et al., 2004). We also found that older children spent significantly more time with the tactile board, but there were no significant correlations with the SCQ, which captured broader autistic behaviours.

### Implications for practice and future research

Current educational practice supports the view that learning is best supported by a child-centred approach (e.g. Nicholas et al., 2021). More specifically, educational practitioners believe MSE use should be centred on the autistic child’s needs, capabilities and preferences (Unwin et al., 2021a). The current findings provide insights that support child-centred MSE use. Focussing MSE use on preferred items has been suggested as a way to improve the efficacy of the MSE and reduce possible discomfort for autistic users (Lancioni et al., 2002; Stephenson & Carter, 2011a). It could also help with session planning, particularly if involving more than one child.

Our evidence suggests that there may be different profiles of preference for MSE equipment. Some children preferred equipment that requires active engagement to produce sensory changes (i.e. tactile board and TSLB). These children were more likely to have higher parent-reported sensory avoidance and sensory sensitivity, lower observed sensory seeking behaviours and higher NVIQ. Other children preferred equipment that provided more dynamic sensory stimulation without requiring active control (i.e. bubble tube). These children tended to have lower parent-reported sensory sensitivity, higher observed sensory seeking behaviours and lower NVIQ. These patterns of engagement would need to be replicated in a real-world MSE environment and a larger cohort. However, our findings provide some initial indications for practitioners of potential equipment preferences of children with different profiles. Practitioners may want to consider using parent- or child-report sensory questionnaires to help inform their educational planning.

An important consideration for practitioners is that MSE equipment can be used flexibly. For example, a TSLB can be programmed into a passive mode, where different colours are displayed in an automatic timed sequence; this would arguably make it more appealing to children who gravitated to the bubble tube. Similarly, ostensibly passive equipment that is preferred by children with a lower NVIQ can be used in a context that provides intellectual stimulation. For example, enabling a child to use a remote-control device to change the bubble tube colour as a way of answering questions.

Practitioners in our previous qualitative study believed that greater enjoyment in the MSE was associated with better relationship building (Unwin et al., 2021a). Although not directly tested, it is likely that enjoyment in the room would be elevated by the use of preferred equipment. Related to this, children with a range of developmental disabilities showed increased prosocial behaviour when preferred equipment, rather than general equipment, was used within the MSE (Fava & Strauss, 2010). A counter to this is that high preference sensory stimulating toys reduced social interactions between autistic children and their typically developing sibling, in contrast to traditional toys that were low in sensory appeal (Sautter et al., 2008). Indeed, autistic adults have reported difficulty in disengaging from an enjoyable sensory experience (MacLennan et al., 2022) and practitioners have reported that sensory rooms can lead to fixations with sensory equipment (Unwin et al., 2021a). Therefore, future research should consider the impact of equipment preference in the MSE on a broad range of outcomes, including relationship building and social dynamics during the sessions. Future research could also investigate how stable preferences are over time and how dependent they are on day-to-day fluctuations in arousal state and mood.

The current study focussed on categories of sensory behaviour that were measured in all participants. Previous research has indicated sensory subtypes in autism. The subtypes reflected shared patterns of sensory behaviour that varied according to the frequency of the behaviours (e.g. Ben-Sasson et al., 2008; Tillmann et al., 2020; Uljarević et al., 2016) or to qualitative differences in the types of behaviours (e.g. Hand et al., 2017; Lane et al., 2010, 2014), or a mixture of both (e.g. Ausderau et al., 2016). Future research should explore whether autistic children with different sensory subtypes respond differently within the MSE. We chose to focus on preference for equipment and did not explore the ways in which children chose to engage with the room, which could include the type and amount of play, and the type and amount of interaction with an adult or peers. A more complete exploration of the relation between sensory profiles and sensory subtypes with MSE patterns of use should also consider these important facets of autistic children’s engagement with the MSE.

Finally, it is also worth considering the implications of these findings beyond education. Considerations of the built environment for autistic people commonly suggest that sensory rooms are included as part of best practice (Black et al., 2022). Thus, these findings may be useful to other contexts in which MSEs are provided for autistic children such as community centres, museums, sporting events and concerts.

### Limitations

The current study used the duration and frequency of equipment use as a proxy to infer preference. However, there are other ways to measure child preference, including coding for signs of enjoyment and asking children directly about preference. Future sensory room research should include the autistic voice, with augmentative and alternative communication (AAC) providing inclusive options to help autistic people express preferences, thoughts and feelings about MSEs.

The free-play session took place at the end of a longer study that included two other MSE sessions, an IQ assessment and an ADOS (see Unwin et al., 2021b). Although breaks were provided, it is possible that fatigue may have affected the children’s behaviours and choices. Furthermore, the tactile board was not used in the longer study and children may have preferred the equipment that was familiar. The 5-min free-play duration used in the study was relatively short, which may not account for changes in preference over time. However, the MSE was novel to all children so general familiarity with the room was controlled. Finally, although the four pieces of equipment assessed in this study included some of the most popular MSE items (Unwin et al., 2021a), the application of our findings are limited to practitioners who have similar equipment.

While we have established preferences of MSE equipment for our sample of autistic children, the initial selection of equipment for our MSE was based on commonly used equipment in special schools (Unwin et al., 2021a), and we did not consult with autistic children directly. Co-creation and co-design with stakeholders has been increasingly recognised as important (Sanders & Stappers, 2008) and the co-design of sensory spaces with autistic people would support their effectiveness and appropriateness. The effect of stakeholder consultation on the acceptability and positive impact of MSEs is an avenue for future research and evaluation.

## Conclusion

In the first investigation of its kind, we found that autistic children’s preferences for equipment within an MSE were related to parent-reported and observed sensory behaviours and NVIQ, but not broader autistic behaviours. At a group level, the children preferred to spend time with the sensory stimulating bubble tube and TSLB. Practitioners working with autistic children in an MSE or seeking to install a new MSE should consider children’s individual characteristics when planning sessions and selecting equipment. Although these findings require replication, they are an important step in developing evidence-based guidelines for MSE use.

## Supplemental Material

sj-docx-1-aut-10.1177_13623613231180266 – Supplemental material for Patterns of equipment use for autistic children in multi-sensory environments: Time spent with sensory equipment varies by sensory profile and intellectual ability Supplemental material, sj-docx-1-aut-10.1177_13623613231180266 for Patterns of equipment use for autistic children in multi-sensory environments: Time spent with sensory equipment varies by sensory profile and intellectual ability by Katy L Unwin, Georgina Powell, Alice Price and Catherine RG Jones in Autism
