# Supplementary material for: Patterns of equipment use for autistic children in multi-sensory environments: Time spent with sensory equipment varies by sensory profile and intellectual ability
Source: Autism. 2023 Jul 7;28(3):644–55. doi: 10.1177/13623613231180266 (PMC10913295; doi:10.1177/13623613231180266)
Supplement: sj-docx-1-aut-10.1177_13623613231180266 – Supplemental material for Patterns of equipment use for autistic children in multi-sensory environments: Time spent with sensory equipment varies by sensory profile and intellectual ability [file sj-docx-1-aut-10.1177_13623613231180266.docx]

**Supplementary materials**

**Supplementary materials 1**

Sensory Profile (SP; Dunn, 1997) quadrants include a range of items that cover different modalities, as well as sensory modulation, behavioural and emotional responses, and behavioural outcomes. The Registration quadrant had two items representing the auditory modality but no visual or tactile modality items. The Avoiding quadrant had three auditory, four visual and three tactile items. The Sensitivity quadrant had two auditory, one visual, and six tactile items. Post-hoc analyses explored the significant correlations for Registration, Avoiding and Sensitivity (see Table 5 in the manuscript) to see if these were driven by particular modalities (Supplementary table 1).

**Supplementary table 1*.*** *Post-hoc exploration of the significant correlations between the duration (s) of time spent at the equipment and Sensory Profile (SP; Dunn, 1999) quadrant scores. Analysis focussed on modality-specific effects (auditory, visual, tactile). There were no visual and tactile modality items for the Registration quadrant. NB. Lower SP scores indicate more sensory symptoms.*

|  | df | Touch, sound and light board | Bubble tube | Tactile board |
| --- | --- | --- | --- | --- |
| **SP: Registration** | | | | |
| Auditory | 39 | - | - | -.24 |
| **SP: Avoiding** | | | | |
| Auditory | 39 | -.09 | - | -.24 |
| Visual | 39 | -.19 | - | -.25 |
| Tactile | 39 | -.28 | - | -.39* |
| **SP: Sensitivity** | | | | |
| Auditory | 39 | - | .12 | -.40* |
| Visual | 39 | - | .33* | -.22 |
| Tactile | 39 | - | 40* | -.47** |

**p<.05, **p<.01.*

Higher parent-reported avoidance (e.g. reacts negatively to touch) and sensitivity (e.g., sensitivity to fabrics) to tactile stimuli was associated with more time at the Tactile board, as was higher levels of auditory sensitivity (e.g. distracted by noise). However, there were no significant correlations with the visual items. For the Bubble tube, higher parent-reported sensitivity to both tactile and visual (bothered by bright lights) was associated with less time spent at the tube. Overall, modality effects were most clear for the Sensitivity quadrant.

**Supplementary materials 2**

**Supplementary table 2*.*** *Spearman’s Rho correlations between the duration Sensory Profile (SP; Dunn, 1999) quadrant scores and the duration of observed sensory seeking behaviours with non-verbal IQ (NVIQ)*

|  | df | NVIQ |
| --- | --- | --- |
| **SP: Quadrant scores** | | |
| Registration | 33 | -.07 |
| Avoiding | 33 | -.14 |
| Seeking | 33 | .12 |
| Sensitivity | 33 | -.22 |
| **Observed sensory behaviours: Duration (s)** | | |
| Seeking | 39 | -.60*** |

References

Dunn, W. (1999). *Sensory Profile: User’s manual*. Psychological Corporation.
